# Intimate partner violence in pregnancy among antenatal attendees at health facilities in West Pokot county, Kenya

**DOI:** 10.11604/pamj.2017.28.229.8840

**Published:** 2017-11-15

**Authors:** Isaac Ogweno Owaka, Margaret Keraka Nyanchoka, Harryson Etemesi Atieli

**Affiliations:** 1 Masters of Public Health (Reproductive Health Program), Kenyatta University, Nairobi, Kenya; 2 Chairperson Department of Environmental and Population Health, School of Public Health, Kenyatta University, and Nairobi, Kenya; 3 Senior Lecture, Department of Public Health, Maseno University, Kenya

**Keywords:** Intimate partner violence, pregnancy, prevalence

## Abstract

**Introduction:**

The objective of this study was to investigate factors contributing to intimate partner violence in pregnancy among antenatal attendees at the health facilities in West Pokot Sub-County. The study was done in West Pokot Sub-County.

**Methods:**

Using cross sectional study design, a total of 238 antenatal attendees were systematically sampled for the study. Four focused group discussions and 20 key informant interviews were conducted for qualitative data collection. Qualitative data was consolidated into various themes while bivariate and logistic regression analysis was done to determine factors associated with experience of IPV in the index of pregnancy with P ≤ 0.05 being considered significant.

**Results:**

The study found prevalence of overall, physical, psychological and sexual IPV in pregnancy to be 66.9%, 29.9%, 55.8% and 39.2% respectively. After adjusting for confounders, Overall IPV in pregnancy was significantly associated with Alcohol intake by partner (OR 2.116, 95% CI 1.950-2.260, P 0.000) and partner's level of education (OR 1.265, 95% CI 1.079-1.487, P 0.031), while psychological and sexual IPV was significantly associated with age of partner (OR 2.292, 95% CI 2.123-2.722, P 0.007) and age of pregnant women (OR 1.174, 95% CI 1.001-1.397 P 0.049) respectively. The care offered to antenatal attendees experiencing IPV was not in line with WHO guidelines and standard on handling gender based violence cases.

**Conclusion:**

The study finding indicates that IPV in pregnancy among antenatal attendees in West Pokot is very high. This unearths the gaps on gender based violence interventions in the maternal and child health programs.

## Introduction

Violence against women perpetuated by intimate partners is a worldwide and an important public health concern as well as human rights issue. More than 1.3 million people worldwide die each year as a result of violence in all its forms, accounting for 2.5% of global mortality [1]. Global estimates of intimate partner violence perpetuated by men against women indicate that 30% of ever partnered women worldwide have experienced physical and/or sexual violence by an intimate partner at some point in their lives [2]. It is also estimated that 15% and 71% of women experience IPV during their lifetime [3]. This kind of violence by intimate partner in pregnancy is manifested by physical, sexual, emotional abusive act and controlling behavior. The prevalence of IPV during pregnancy in sub-Saharan Africa is among the highest reported globally [4]. In Kenya, it is estimated that 38% of women suffer from IPV in their lifetime [5]. While gender based violence towards pregnant women in Kenya is estimated to be 13.5% [6], a higher prevalence than that of many conditions normally screened for during pregnancy [7]. Pregnant women exposed to violence are more likely to experience stress, depression, miscarriage, pre-term delivery, induced abortion, and stillbirth [8]. Their infants are in turn, more likely to experience low birth weight, illness, under-nutrition and mortality [9]. This study represents an important step toward redressing the dearth of evidence on the prevalence intimate partner violence and the quality of care in pregnancy both the health facility and community level in the nomadic and pastoralist community of West Pokot County.

## Methods

**Study area:** The study was conducted in health facilities in West Pokot Sub-County in West Pokot County, Kenya. It lies within longitude 34°47' and 35°35' East and latitude 1 and 2 North.

**Research design:** This was both quantitative and qualitative descriptive cross-sectional study to determine the prevalence of intimate partner violence during the pregnancy among antenatal attendees in 11 sampled health facilities.

**Sampling procedure:** Stratified two stage random sampling was used in this study. The health facilities in the Sub-County were first stratified according to MOH service delivery levels (Republic of Kenya/MOH/Health Secretariat, 2005). This resulted into three strata; Level II, III and IV from which 11 health facilities were proportionately and randomly sampled from a total of 20 health facilities in the Sub-County. The respondents were distributed proportionately to each health facility depending on the average number antenatal attendees in health facilities in the year 2012 as per District health information system .This was followed by systematic random sampling of 238 participant at interval of three mothers at each health facility to identify the respondents where the interval was the overage monthly antennal attendance divided by required sample size (622/238 = 2.613≈3). The starting point was randomly generated number at each facility included in the study. This was done using the established clinic queues to systematically sample antenatal attendees for interview until the sample size was achieved for each health facility. Data was collected within one month from 1^st^ to 26^th^September 2014 to avoid any bias that might have resulted from antenatal revisits in the following month.

**Data analysis:** Data was entered; cleaned, analyzed and stored using SPSS. Descriptive analysis of variables and graphical presentation was done using proportions and frequency to describe the social demographic characteristics of women and intimate partner's. Prevalence of women reporting the various forms of IPV in the current pregnancy was sought. Odd ratio was used to show the strength of associations. Bivariate analysis was done to compare independent factors of women who experience violence in the index of pregnancy with women who did not. Multiple logistic regression analysis predicting overall, physical, psychological and sexual IPV was used to explore the adjusted association of covariates that had a p < 0.1 in the bivariate analyses with a P ≤ 0.05 being considered significant. Responses from the FGDs and KIIs were analyzed by content analysis, summarized under various themes, inferences made from each theme and conclusions drawn was then triangulated with the data from the questionnaire.

## Results

**Sociodemographic characteristics of respondents:** The study approached 238 antenatal attendees and achieved a response rate of 224 (94.1%). Among the 224 women respondents, 44 (19.6% were aged between 35 and 39), 20 (8.9%) were aged between 45-49. More respondents reported to have had more than one pregnancy 117 (52.2%) while 311 (13.6%), 90 (40.2%) and 103(45%) were in their first, second and third trimesters respectively. Most respondents reported to be married 184 (82.1%) with 116 (51.8%) having been in marriage for four year and less and only 65 (29%) had been married for ten years and above. Most of women had formal education 182 (81.3%) with the highest level of complete primary school 114 (50.9%). The majority of the respondents 168 (75%) were unemployed.

**Prevalence of intimate partner violence:** The prevalence of overall, physical, psychological and sexual IPV was in the current pregnancy was 150 (66.9%), 67 (29.9%), 125 (55.8%), 88 (39.2%) respectively. Figure 1 is a three-way contingency table analysis of different forms of IPV experienced singly or combination. These results were also supported by the outcome of the FGDs. Across all the FGDs participants mentioned psychological, physical, sexual, and economical violence. Psychological was ranked first, Economical second; where the man controls the expenditure of the woman, sexual third and physical fourth .

**Predisposing risk factors of IPV in pregnancy:** The bivariate analysis showed association between various factors and overall IPV among the pregnant women, however only alcohol intake by partner was significantly associated with overall IPV (p = 0.000). Table 1 shows multiple logistic regression analysis to determine factors that were independently associated with overall IPV. This was done while controlling for variables with p ≤ 0.1. Two factors remained significant risk factors of IPV in pregnant women. More violence was reported by pregnant women whose partner were taking alcohol, (p = 0.000). Women whose partner had no education were also more likely to experience overall IPV, (p = 0.031).

**Table 1:** Multiple logistic regression analysis of risk factors for overall IPV

Independent Variables	B	S.E.	p-value	OR	95% C.I. for OR	
					Lower	Upper
Age of respondent	-0.289	0.375	0.441	0.749	0.359	1.562
Age of partner	-0.514	0.462	0.266	0.598	0.242	1.478
Length of relationship	0.095	0.207	0.646	1.100	0.733	1.650
Partner’s level of education	-1.329	0.617	0.031*	1.265	1.079	1.487
Partner drinking alcohol	-2.156	0.413	0.000*	2.116	1.950	2.260

**Risk factors associated with physical violence:** Level of education of pregnant women, polygamy marriage and alcohol intake by both partner and pregnant women were significantly associated with physical IPV in bivariate analysis (p ≤ 0.05). Table 2 shows multiple logistic regression analysis of factors that were associated with physical violence. Only alcohol intake by partner remained significantly associated with physical IPV, (p = 0.000). Pregnant women with partner who drinks alcohol were three times more likely to experience physical violence than pregnant women whose partner does not drink alcohol.

**Table 2:** Multiple logistic regression analysis of risk factors for physical IPV

Independent Variables	B	S.E.	p-value	OR	95% C.I. for OR	
					Lower	Upper
School attendance (respondent)	1.354	0.934	0.147	0.873	0.621	1.139
Respondent level of education	-0.306	0.237	0.197	0.736	0.463	1.172
Polygamy	-0.343	0.432	0.426	0.709	0.304	1.653
Respondent taking alcohol	-0.153	0.486	0.754	0.858	0.331	2.225
Partner taking alcohol	-1.600	0.343	0.000*	3.202	3.103	3.395
Constant	1.590	1.353	0.240	0.902		

**Risk factors associated with psychological violence**: Age of partner, Length of relationship and Partner's alcohol intake were significantly associated with psychological IPV, (P < 0.05) in the bivariate analysis while Multiple logistic regression analysis indicated two factors to be associated with psychological IPV. Pregnant women with partner less than 25 years of age were two time more likely to experience psychological IPV than women whose partner were more than 25 years of age (p = 0.007) while pregnant women with partner who takes alcohol were slightly more likely to experience psychological IPV than those whose partner don't take alcohol (p = 0.000) (Table 3).

**Table 3:** Multiple logistic regression analysis of risk factors for psychological /emotional IPV

Independent Variables	B	S.E.	p-value	OR	95% C.I. for OR	
					Lower	Upper
Age of respondent	-0.108	0.337	0.749	0.898	0.463	1.739
Age of partner	-1.210	0.451	0.007*	2.298	2.123	2.722
Length of relationship	0.127	0.190	0.505	0.135	0.082	0.348
Partner taking alcohol	-1.337	0.316	0.000*	1.263	1.142	1.487
Constant	3.788	1.074	0.000	44.189		

**Risk factors associated with sexual violence:** Partner's level of education and partner alcohol intake were significantly associated with sexual IPV (P < 0.05) while age of respondent was not significantly associated with sexual IPV (P = 0.065) in bivariate analysis. However multiple logistic regression analysis indicated only alcohol intake by partner to be significantly associated with sexual IPV, (P = 0.015). Pregnant women with partner's who take alcohol were 2.486 more likely to experience sexual IPV than pregnant women whose partner were not taking alcohol (Table 4).

**Table 4:** Multiple logistic regression analysis of risk factors for sexual IPV

Independent Variables	B	S.E.	p-value	OR	95% C.I. for OR	
					Lower	Upper
Partner’s level of education	-0.555	0.281	0.049*	1.174	1.001	1.397
Partner school attendance	-1.106	0.607	0.069	0.931	0.701	1.988
Partner taking alcohol	-0.722	0.287	0.012*	2.486	2.277	2.852
Constant	2.816	0.987	0.004	16.708		

## Discussion

This study found overall IPV in the current pregnancy to be 66.9% among the antenatal attendees, which is the highest in African countries but close to 63.1% reported in Zimbabwe [10]. A review of 19 studies conducted in Africa showed overall IPV prevalence during pregnancy to be ranging from 2.3% in Nigeria to 57.1 in Uganda and a meta-analysis yielded an overall prevalence of 15% (95% CI = 14-16%) [4]. This prevalence is also much higher than gender based violence toward pregnant women in Kenya which is estimated to be 13.5% [6] and overall IPV in the index of pregnancy as reported to be 37% in the Kisumu study [11]. However, it is within the global range of lifetime IPV in the general women population of 15% to 71% [3]. The wide ranging estimates could be attributed to social and cultural diversity in Africa [12]. Furthermore, only three of the nineteen African reviewed studies determined psychological IPV in pregnancy hence lower overall IPV as compared to this study which included all the three forms of IPV. This study found the prevalence of physical IPV in the current pregnancy to be 29.9% this is within the range of three various studies conducted in Africa which found physical IPV during pregnancy to be ranging from 22.5% to 40% [4], but higher than a facility based study conducted in Kisumu, Kenya that found prevalence of physical IPV during pregnancy to be 10% [11]. The variation estimates are likely to be as a result of different social and cultural context. The study showed emotional/psychological IPV prevalence of 55.8% which is lower than the prevalence of 66.2% reported by a study conducted in Abeokuta, Nigeria among antenatal attendees in three health facilities [13].

However, this result is higher than a study conducted at Kisumu District Hospital where the prevalence of emotional/psychological IPV during pregnancy was found to be 29% [11] and also above the range of three African studies that reported IPV during pregnancy to be between 24.8% and 49% [4]. Additionally, this study revealed sexual IPV in the current pregnancy to be 39.2% which is higher than other studies conducted in Africa that reported prevalence of sexual IPV during pregnancy to be 2.7% in Uganda, 12% in Kenya, 26.5 % in Nigeria and 19% in South Africa and 38.9 in Zimbabwe [11, 14-16]. This high prevalence of sexual IPV in the study setting may be due to cultural reasons that, the initiator for sex is usually the male partner as women are shaped to satisfy their partners. To some extent, sex has remained a silent subject of discussion in most pastoralist communities even between intimate partners where women are not expected to express their desire. This prevailing societal norm put men as the determinant of sexual affairs in any relationship and women must just comply. Generally, variation in the violence prevalence estimates may be due to true differences in the prevalence of violent acts within different study populations, as well as methodological differences between studies [17]. The prevalence rates could also be because this study was clinic based but more notably questions referred to the current pregnancy at any of the gestation ages which potentially reduced recall bias. Most other studies collected data in the 1^st^, 2^nd^ or 3^rd^ trimesters of the most recent or previous pregnancy unlike this study that asked questions specific to current pregnancy. These results on prevalence were also supported by the outcome of the FGDs where Psychological IPV was ranked first, Economical second; where the man controls the expenditure of the woman, sexual third and physical forth. Parity, marital status, gestation age, level of education of pregnant women and occupation of both pregnant women and partners were not associated with overall, physical, psychological and sexual IPV in pregnancy before and after controlling for confounding and interaction between independent factors which differ from other studies.

A study in Abeokuta Nigeria revealed low level of education to be significantly associated with experiencing violence during pregnancy [13]. Inconsistently, a study among antenatal attendees at the University of Port Harcourt teaching hospital found experience IPV to be of more in women of low parity [18]. Unlike this study, other studies have found that women are more likely to experience violence during pregnancy if they are unmarried [19,20]. Length of current relationship, level of education and polygamous marriage were associated with various IPV but were not significant after controlling for confounding and interaction between independent factors, (p > 0.005). This result is inconsistent with a study conducted in Kisumu District Hospital which reported polygamy to be significantly associated with overall IPV in pregnancy [11] but in agreement with the same study that there is no significant association between IPV and level of education. Project involving more than 1000 pregnant women in the US revealed that income and education levels were the most significant predictors of pregnancy violence [21]. However, a systematic review of African studies identified three studies that reported strong positive association between pregnant women's low level of education and experiencing IPV and (p < 0.005) and six other studies where the relationship did not reach statistical significance (p ≤ 0.005) [4]. Alcohol intake by partner was found to be significantly associated with overall, physical, psychological and sexual IPV in pregnancy. This finding is consistent with findings from other five studies conducted in Africa [4]. The relationship between IPV and alcohol use is complex because it can be bidirectional with alcohol drinking leading to IPV or IPV leading alcohol drinking or it may involve both partner [22]. Alcohol use can also result in household neglect facilitating marital or relationship tension that may result to violence [23].

This study also found that women whose partner had not attended school were more likely to experience overall IPV. Other research findings have revealed that men who perpetrate violence during pregnancy tend to have lower socioeconomic status. This includes lower levels of education [24]. Most studies have only reported significant relationship of level of education and IPV in pregnancy but not school attendance only as reported in this study. Age of pregnant women and Age of the partner were also found to be risk factors to psychological and sexual IPV in pregnancy respectively. Pregnant women aged 25 years and below were more likely to experience sexual IPV, while pregnant women with partner aged 25 years and below were more likely to experience psychological IPV. This finding is consistent with a number of studies. Other studies have also documented younger age among pregnant women be associated with increased IPV [25]. Two studies estimated that women younger than 20 years old were three to four times more likely than women aged 30 or older to experience violence during pregnancy [19,20]. Additional risk factors identified during the FGDs were pregnancy itself and cultural norms. The study identified that unwanted or unintended pregnancy could be a source of intimate partner violence. This in agreement with study which indicated that expecting a first child as well as unplanned or unwanted pregnancy is a significant risk factor for IPV [17]. One participant highlighted that most women are always quarrelsome, moody easily irritated resulting into IPV, this is explained by some studies have indicated that violence is likely to escalate during pregnancy or pregnancy triggers violence [26]. The physical and emotional changes in women during pregnancy brings with them some demands for more or different economic, social and sexual requirement in a partnership which normally place pressure on men hence trigger violence [27]. Many participants described IPV as a consistent and unchangeable aspect of local culture. This concurs with a study conducted in Kenya which indicated that men are socialized to believe that they are superior to women, should dominate their partners and endorse traditional gender roles and Women's subordination and submission is then considered to be normal, expected, accepted [28]. Moreover a study in Ethiopia also found several community members believed IPV against women was acceptable under particular conditions including failure to give birth, suspicion of infidelity, constatntly arguing with the husband or neighbor/community member, disobeying her husband and circumstances in which a woman attempts to go against the culture and vocalize her thoughts or opinion [29].

## Conclusion

The overall, physical, psychological and sexual IPV of 67%, 30%, 56% and 40% respectively during pregnancy among antenatal attendees in this study are among the highest ever reported in a health facility based study. The major risk factors for IPV were alcohol intake, age of both women and partner's and lack of formal education by partner. All the three forms of IPV were associated with partner's alcohol intake, while sexual, psychological and overall IPV were associated with age of antenatal attendees, age of partners and level of education of partners respectively. The quality of health care services offered to antenatal attendees experiencing violence was not in line with WHO guidelines and recommendations on advocacy, social mobilization, screening, documentation, referral system and case management procedures. The results demonstrated inadequate skills and knowledge among the health workers on handling IPV victims, lack of screening of for IPV, no referral network, inadequate documentation of IPV cases and unconducive environment for communication of IPV issues. In light with the study findings, the Ministry of health and other partners should promoteintegration of IPV screening services in Maternal and child health clinic with aim identifying those experiencing violence and also identifying those at risk due to their own and partners' sociodemographic characteristics to initiate timely preventive measures. The health workers skills and knowledge can be improved through continuous sensitization of health workers through training in gender based violence to enable them recognize and respond to high IPV risk cases. At the community level, the Ministry of health should implement primary prevention interventions in form of community educational campaigns to sensitize the community on maternal and child health consequences of IPV in pregnancy and together with the community leaders address inequitable norms, beliefs and practices, secondary prevention mechanisms by health workers in antenatal and post-natal care settings should address IPV during pregnancy because these are unique opportunities to consistently contact women at risk. Finally, wide dissemination to increase national and public awareness on the high prevalence of IPV in pregnancy and screening for IPV among pregnant women visiting antenatal health care facilities in West Pokot may enable implementation of appropriate interventions among abused pregnant women.

### What is known about this topic

- Estimated 38% of women in Kenya suffer from IPV in their lifetime;
- Gender based violence towards pregnant women in Kenya is estimated to be 13.5%, (Drivers & Johnson, 2010) a higher prevalence than that of many conditions normally screened for during pregnancy.

### What this study adds

- Risk factors of IPV in pregnancy among antenatal attendees;
- Quality of care offered to pregnant women experiencing intimate partner violence.

## Competing interests

The authors declare no competing interest.
